# *Dracula aguilarii* (Orchidaceae, Pleurothallidinae), a new species from southwestern Ecuador and the first confirmed location for *Dracula soennemarkii*

**DOI:** 10.3897/phytokeys.273.182821

**Published:** 2026-04-23

**Authors:** Luis E. Baquero R., Marco M. Jiménez, Gabriel A. Iturralde

**Affiliations:** 1 Grupo de Investigación en Biodiversidad, Medio Ambiente y Salud BIOMAS, Carrera de Ingeniería Agroindustrial, Facultad de Ingenierías y Ciencias Aplicadas, Universidad de Las Américas, Vía a Nayón, Quito 170124, Ecuador Universidad de Las Américas Quito Ecuador

**Keywords:** Biodiversity, cloud forest, habitat fragmentation, premontane forest, taxonomy

## Abstract

The genus *Dracula* comprises more than 130 species of epiphytic orchids, with its highest diversity in the northern Andes of Colombia and Ecuador. Here, we describe *Dracula aguilarii***sp. nov**. from El Oro Province in southwestern Ecuador. The novelty resembles *D. hirtzii* in its large, cream-colored flowers suffused with brown, and shortly pubescent sepals, but differs in having narrowly obovate, obtuse leaves, a deep, broad, rounded sepaline cup, and shorter sepaline tails. We also report the first confirmed locality for *D. soennemarkii*, previously described without collection data. Preliminary conservation assessments based on IUCN Red List criteria indicate that *D. aguilarii* is Endangered (EN) and *D. soennemarkii* is Critically Endangered (CR) due to their extremely restricted distributions and ongoing habitat loss.

## Introduction

The genus *Dracula* Luer (Pleurothallidinae) comprises more than 130 species characterized by a distinctive floral morphology, including conspicuous membranous to fleshy sepals connate into a flat or cupped synsepal bearing elongate tails, reduced oblong petals with a bivalved apex, and a lip differentiated into a hypochile and a rounded, concave epichile with radiating lamellae (Luer 1978, 1986, 1993; Luer and Thoerle 2010). Molecular phylogenetic studies place *Dracula* as sister to *Masdevallia* although *Dracula xenos* Luer & R.Escobar has been shown to fall within *Masdevallia* (Pridgeon et al. 2001; Meyer and Cameron 2009; Faria et al. 2025).

Species of *Dracula* are pollinated by flies of the genus *Zygothrica* Wiedemann, 1830. The lip mimics the odor and lamellar structures of macrofungi, and brood-site imitation has been proposed as the primary pollination strategy, whereby flies inadvertently effect pollination while attempting oviposition (Endara et al. 2010; Gil-Amaya et al. 2025). However, a recent study of *Dracula erythrochaete* did not document oviposition events or egg deposition during prolonged floral visits, leaving aspects of the pollination mechanism unresolved (Gil-Amaya et al. 2025).

Species diversity of *Dracula* is concentrated in the northern Andes, particularly in Colombia and Ecuador, the latter harboring 62 species (POWO 2026).

Lower montane rainforests of Ecuador are recognized for their high density and diversity of vascular epiphytes, although comprehensive quantitative assessments are still lacking (Jørgensen and Léon-Yánez 1999). El Oro Province, in southwestern Ecuador, comprises 19 ecosystems, distributed along an elevational gradient from sea level to 3,900 m. These Andean premontane and cloud forests support a rich epiphytic flora, with orchids representing a prominent component (Yánez-Muñoz et al. 2019). Remnant native forests harbor endemic species of *Dracula*, including *D. cordobae* Luer, *D. mopsus* (F. Lehmann & Kraenzlin) Luer, *D. woolwardiae* (F. Lehmann ex Kraenzlin) Luer, and the natural hybrid *D. × pinasensis* Zambrano & Solano.

Despite the extensive taxonomic work in *Dracula*, new species continue to be discovered (Doucette 2011; Doucette 2012; Luer and Thoerle 2012; Meyer et al. 2012; Baquero 2013; Cavestro 2013; Baquero and Meyer 2014; Cavestro and Fernández 2016; Peláez et al. 2020; Baquero et al. 2025), and some taxa remain poorly known or lack precise locality data (Luer 1993; Parra-Sanchez and Baquero 2023). One such case is *Dracula soennemarkii* Luer & Dalström originally described without a specific locality (Luer and Thoerle 2012). Since its publication, the distribution of this species has remained uncertain, and no confirmed wild populations have been documented. Nevertheless, the species is cultivated, commercially traded and exported and is well-known among *Dracula* growers. Here we document, for the first time, a confirmed locality for *D. soennemarkii*.

Prior to 2011, near the locality where we report *D. soennemarkii*, Mario Aguilar discovered a population of approximately 15 individuals in a remnant cloud forest near the town of Balsas, with a unique combination of morphological characters not matching any previously described taxon. Subsequent field observations and herbarium work corroborated that these plants represented an undescribed species. A second population of this novelty was later located approximately 13 km north, where a single flowering individual was confirmed (Figs 3, 6). A third locality was recently documented approximately 4 km south of Balsas by José Bustamante, a local orchid enthusiast (Fig. 7). Here, we describe and illustrate this new species of *Dracula* thereby contributing to a better understanding of species diversity within the genus and highlighting the conservation importance of the premontane and cloud forests of southwestern Ecuador.

## Materials and methods

### Study area and permits

Fieldwork was conducted in El Oro Province, southwestern Ecuador, within premontane and cloud forest remnants at 900–1200 m elevation. Material of the new species was collected under Research Permit No. MAATE-DBI-CM-2025-0458 issued by the Ministerio del Ambiente, Agua y Transición Ecológica (MAATE).

### Taxon sampling, voucher preservation and morphological analysis

Specimens of the new species and *Dracula soennemarkii* were documented both in situ and ex situ. Photographs of floral and vegetative structures were taken with a Nikon D5100 + AF-S Micro Nikkor 60 mm, and a Panasonic FZ300 equipped with a Raynox DCR-150 macro adapter. Figures were prepared using Adobe Photoshop 2019 (Adobe Systems, San Jose, CA, USA). Voucher specimens were deposited in the herbarium of the Instituto Nacional de Biodiversidad, Museo Ecuatoriano de Ciencias Naturales (QCNE). Fresh flowers were preserved in 70% ethanol with 1% glycerol.

Living plants were examined and compared with morphologically similar species, incorporating observations from preserved specimens and information from published sources (e.g., Luer 1993, 1994, 2002, 2005, 2009; Luer and Thoerle 2010, 2012). Images of relevant type specimens were reviewed via JSTOR Global Plants (https://plants.jstor.org; accessed 25 July 2025), Tropicos (https://tropicos.org; accessed 25 July 2025), and iDigBio (https://www.idigbio.org; accessed 25 July 2025). No additional records of the studied species were found on iNaturalist (https://www.inaturalist.org/). Botanical terminology followed Beentje (2016), including color standards.

### Distribution mapping and conservation assessment

Georeferenced occurrence data were compiled to generate a distribution map in QGIS 3.40.11-Bratislava (https://qgis.org/). These data were subsequently used to calculate the extent of occurrence (EOO) and area of occupancy (AOO) with GEoCAT (Bachman et al. 2011), applying a 2-km grid (cell area = 4 km^2^) in accordance with IUCN Red List Guidelines (IUCN 2024), to perform a preliminary conservation assessment. Since the estimated EOO of the new species (4.2 km^2^) is smaller than AOO, it was rounded to equal the latter, according to the IUCN Red List guidelines. The EOO of *D. soennemarkii* cannot be calculated because there is a single occurrence. Geographic coordinates of the specimens are omitted in the text for conservation reasons; detailed data can be consulted in the herbarium vouchers.

## Taxonomic treatment

### Dracula aguilarii

Taxon classificationPlantaeAsparagalesOrchidaceae

Baquero, M.M.Jiménez & Iturralde sp. nov.

7038ABE0-8481-5EC6-89AB-F6400F8D9BCE

urn:lsid:ipni.org:names:77379002-1

Figs 1, 2, 3

#### Diagnosis.

*Dracula aguilarii* is most similar to *Dracula hirtzii* Luer in having large, cream-colored, suffused with brown-reddish, and sepals with acute apices, but differs by its narrowly obovate, obtuse leaves (*vs*. elliptic, acute), densely pubescent sepals (*vs*. microscopically pubescent to glabrous), a broad, rounded, concave sepaline cup that includes the mentum and extends from the connate sepals onto the base of the dorsal sepal (*vs*. narrow, longitudinal mentum without additional sepal depression), shorter sepaline tails, the transversely elliptic hypochile (*vs*. oblong), and the smaller, transversely subquadrate epichile (Table 1, Figs 4, 5).

**Table 1.** Comparison of *Dracula aguilarii* and related species (*D. hirtzii*, *D. ligiae*, and *D. roezlii*).

	*Dracula aguilarii*	*Dracula hirtzii*	*Dracula ligiae*	*Dracula roezlii*
**Plant size**	30 cm	20 cm	20 cm	25 cm
**Leaf shape**	narrowly obovate, obtuse	elliptic, acute	obovate, acute	ovate, acute
**Leaf length**	20–27 cm	12–18 cm	15–27 cm	15–25 cm
**Leaf width**	3–4 cm	2.0–3.5 cm	3.0–4.5 cm	2.5–5.5 cm
**Peduncle**	descending, verrucose	horizontal, sparsely bracted	horizontal, sparsely bracted	horizontal, sparsely bracted
**Inflorescence type**	congested, successively flowered raceme	lax raceme	lax raceme	lax raceme
**Dorsal Sepal shape**	ovate, concave	ovate, flat	broadly ovate, shallowly cupped	ovate, broad, flat
**Sepal tail length**	3.4–6.0 cm	7–10 cm	2.5–3.0 cm	7–8 cm
**Petal shape**	subpandurate, papillose	oblong-spathulate	oblong, papillose	oblong, papillose
**Epichile shape**	subquadrate, concave	suborbicular, concave	ovoid, deeply concave	ovoid, deeply concave
**Geographic distribution**	Ecuador (El Oro)	Ecuador, Colombia	Colombia	Colombia
**Elevation range**	900–1200 m	1500–2100 m	2050 m	1800–2300 m

#### Type.

Ecuador • El Oro: Near Balsas, 1212 m, 13 April 2017, *L. Baquero 3142* (holotype: QCNE!).

#### Description.

Epiphytic ***herb***, erect, caespitose, large up to 30 cm long. ***Roots*** slender, 0.6 mm in diameter. ***Ramicauls*** erect, slender, 1.0–1.4 cm long, enclosed by 2–3 loose, tubular sheets. ***Leaf*** erect, narrowly obovate, 20–27 cm long (including an indistinct petiole), 3–4 cm wide, thinly coriaceous, carinate abaxially, apex obtuse. ***Inflorescence*** a successively flowering raceme, born on a sparsely bracted, pendent, slightly verrucose peduncle, 15–20 cm long from the ramicauls; floral bract tubular, 8–10 mm long; pedicel 16 mm long. ***Ovary*** green suffused with blood-red, 5 mm long, slightly verrucose, round to slightly ribbed in cross-section; ***Sepals*** cream-colored suffused with brown-reddish toward the margins, densely pubescent, covered by brown-red warts and papillae adaxially, cream-colored with brown-red carinae abaxially; ***dorsal sepal*** ovate, 3.5 × 2.3 cm, connate to the lateral sepals for 9 mm, concave basally and convex towards the apex, apex acute, contracted into an erect, brown-red, slender tail, 3.4–6.0 cm long; ***lateral sepals*** oblique, ovate, 3.6 × 2.2 cm, concave basally and convex toward the apex, connate for 2.3 cm to form a broad, rounded sepaline cup, 8 mm deep, which extends from half of its length from the connate sepals, including the slightly deeper mentum underneath the lip, toward the base of the dorsal sepal; apex acute, contracted into tails similar to the dorsal sepal, 3.5–6.0 cm long. ***Petals*** cream-colored, suffused with orange toward the margins and marked with red-brown at the middle and on the adaxial side of the apex, cartilaginous, subpandurate, 4 × 2.3 mm, apex bivalved, brown-red, obtuse, rounded, papillose between the valves, the inner lamina slightly erose, the outer lamina recurved. ***Lip*** white with rose, suffused with orange, spathulate, 8.9 × 5.2 mm; epichile light rose, transversally subquadrate, 4.6 × 5.2 mm, concave, apex rounded and inflated, margins slightly involute and toothed, with three primary, orange lamellae and multiple, radiating, branching, elevated, rose suffused with orange secondary lamellae perpendicular to the primary lamellae; hypochile transversely elliptic, 4.3 × 2.5 mm, with erect, marginal, rounded angles, cleft centrally, base flexibly hinged to the column-foot, concave. ***Column*** white suffused with yellow, stout, semiterete, 4 mm long with 4 microscopic denticles at the apex. ***Anther cap*** white, 1.5 mm long. ***Pollinia*** 2, yellow, 1 mm long. ***Fruits*** and seeds not observed.

#### Eponymy.

Named for Mario Aguilar, farmer, and orchid enthusiast from El Oro, with an extraordinary talent for finding rare orchids in nature, who first spotted the new species in the field.

#### Distribution and ecology.

*Dracula aguilarii* is currently known from three localities in El Oro Province, southwestern Ecuador, at elevations of 900–1200 m (Fig. 6).

The species grows epiphytically on old, moss-covered trees, from the base of the trunk up to about 4 m above the ground. Nearby, other *Dracula* species such as *D. cordobae* and *D. woolwardiae* were observed (Fig. 8). Several Pleurothallidinae (Orchidaceae) were also found growing sympatrically with *D. aguilarii*, including *Platystele alucitae* Luer, *P. caudatisepala* (C. Schweinfurth) Garay and *Specklinia colombiana* (Garay) Pridgeon & M.W. Chase.

#### Conservation assessment.

*Dracula aguilarii* is known from three locations: the original site near Balsas and two additional sites approximately 4 km and 10 km to the north (Fig. 6). It has an estimated area of occupancy (AOO) and extent of occurrence (EOO) of 12 km^2^ (Fig. 6).

The cloud forests of the El Oro Province represent some of the last remnants of this ecosystem in southwestern Ecuador. According to GAD El Oro (2019), only about 6% of the province retains moderately preserved humid forests. Satellite imagery (Google Earth Pro) and field observations show strong habitat fragmentation, consistent with Hermes et al. (2017), who describe the region as comprising small, isolated forest patches surrounded by extensive agricultural lands and pastures, with most remaining forests being young secondary stands or selectively logged.

All known populations of *Dracula aguilarii* occur in small, highly fragmented forest remnants. The most serious plausible threat defining “locations” is rapid forest clearing for cattle pastures, such that a single clearing event would affect most or all individuals within each remnant patch. Additional threats include illegal harvesting due to a persistent demand for new *Dracula* species among orchid collectors in Ecuador (Yeager et al. 2020).

We recommend classifying *D. aguilarii* as Endangered (EN) under criteria B1ab(iii)+2ab(iii), based on an EOO < 5000 km^2^, AOO < 500 km^2^, number of locations < 5, and inferred decline of quality of habitat (Hermes et al. 2017 and pers. obs.).

#### Additional specimen examined.

**Ecuador** • **El Oro**: Vía a Piñas, 957 m, 14 April 2017, flowered in cultivation at La Chilca, *Luis E. Baquero LB 3143* (QCNE!).

#### The first confirmed locality for *Dracula soennemarkii*.

*Dracula soennemarkii* was originally described in 2012 based on a plant purchased from Ecuagenera, an Ecuadorian orchid nursery, and exported under the name *D. woolwardiae* (Luer and Thoerle 2012). The plant was cultivated by J. Sönnemark in Sweden, and its natural origin remained unknown until now.

We confirm that *Dracula soennemarkii* occurs in El Oro Province, southwestern Ecuador, in the same habitat type as *D. aguilarii*, although not at the same locality. A sterile individual of *D. soennemarkii* was found and collected at the confirmed site and subsequently cultivated ex situ until flowering. Upon anthesis, the ascending inflorescences bearing cupped flowers, shortly pubescent sepals, and a small lip with an indistinctive transition between the hypochile and the epichile, the latter with multiple narrow inward-pointing teeth, unequivocally supported its identification as *D. soennemarkii* (Fig. 8F). The species differs from *D. woolwardiae* in having smaller, less expanded flowers with shorter pubescence and tails of the sepals, and lacking a well-defined transition from the hypochile to the shallower epichile.

### Dracula soennemarkii

Taxon classificationPlantaeAsparagalesOrchidaceae

Luer & Dalström, Harvard Papers in Botany, 17(2): f. 11. 2012.

0CD73288-F760-5C40-AE3F-5863E6F0B9C4

#### Type.

Ecuador, *s. loc*., purchased from Ecuagenera, Gualaceo, Ecuador as *Dracula woolwardiae*, flowered in cultivation at Halmstad, Sweden, J. Sönnemark, July 2011, *S. Dalström 3506* (Holotype: W; Isotype: MO).

#### Specimen examined.

**Ecuador** • **El Oro**: Vía a Piñas, 1271 m, 13 April 2017, *Luis E. Baquero, LB 3138* (QCNE!).

#### Conservation assessment for *D. soennemarkii*.

*Dracula soennemarkii* is known from a single location, located 3 km from the second *D. aguilarii* locality (Fig. 6). Its AOO is 4 km^2^. This species is subject to the same threats described for *D. aguilarii*, especially rapid forest clearing for pastures and illegal harvesting.

We recommend classifying *D. soennemarkii* as Critically Endangered (CR) under criteria B2ab(iii), based on an AOO < 10 km^2^, number of locations = 1, and inferred decline of quality of habitat (Hermes et al. 2017 and pers. obs.).

## Discussion

Molecular studies of *Dracula* (Meyer and Cameron 2009; Faria et al. 2025), together with and ongoing phylogenomic analysis by the first author, indicate that molecular evidence currently provides limited resolution at the species level within the genus. At most, these data support the recognition of major clades, particularly the basal lineages, but do not reliably discriminate between species.

Morphologically, *Dracula aguilarii* is most similar to *D. hirtzii*, *D. roezlii* (Rchb.f.) Luer, and *D. ligiae* Luer & R.Escobar. It is readily distinguished by a unique combination of characters, including a very long, descending, slightly verrucose peduncle; comparatively short sepaline tails; an ovate dorsal sepal concave at the base; and, most diagnostic, a deep, broadly rounded sepaline cup incorporating the mentum and extending onto the connate lateral sepals and base of the dorsal sepal. The sepaline cup is at least twice as wide as the lip, contrasting sharply with the narrow, longitudinal synsepaline concavity present in the most similar species. Additional diagnostic features include a subquadrate, concave epichile. This coherent set of floral traits, together with its occurrence at lower elevations in southwestern Ecuador, supports the recognition of *D. aguilarii* as a distinct species (see Table 1 for detailed morphological comparisons).

**Figure 1.**
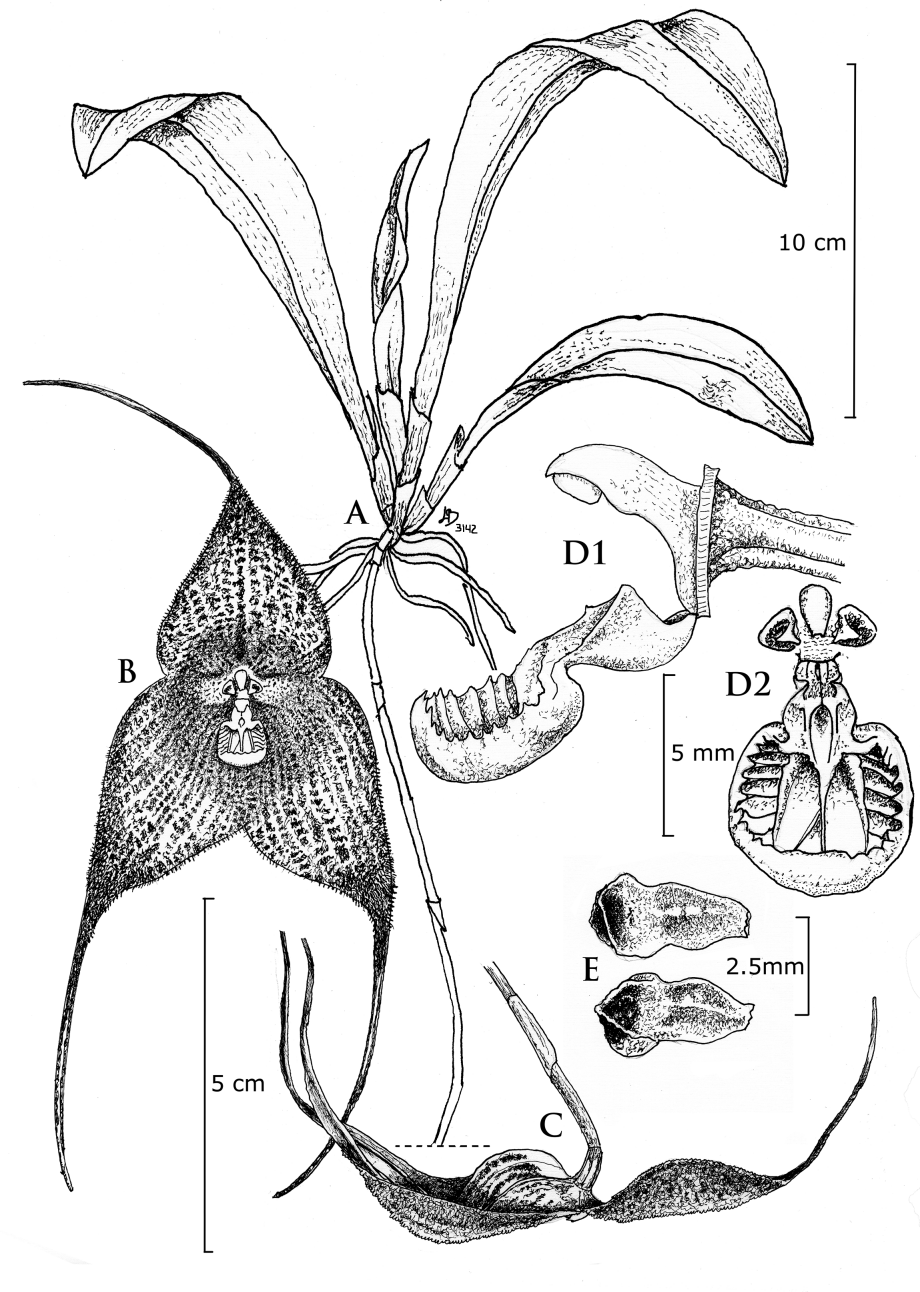
Overview of *Dracula aguilarii* Baquero, M.M. Jiménez & Iturralde. **A**. Habit; **B**. Frontal view of the flower; **C**. Side view of the flower; **D1**. Lateral view of the ovary, column and lip; **D2**. Frontal view of the column, petals, and lip; **E**. Petals, adaxial and abaxial views. Illustration by Luis E. Baquero, based on the holotype (*LB 3142*).

**Figure 2.**
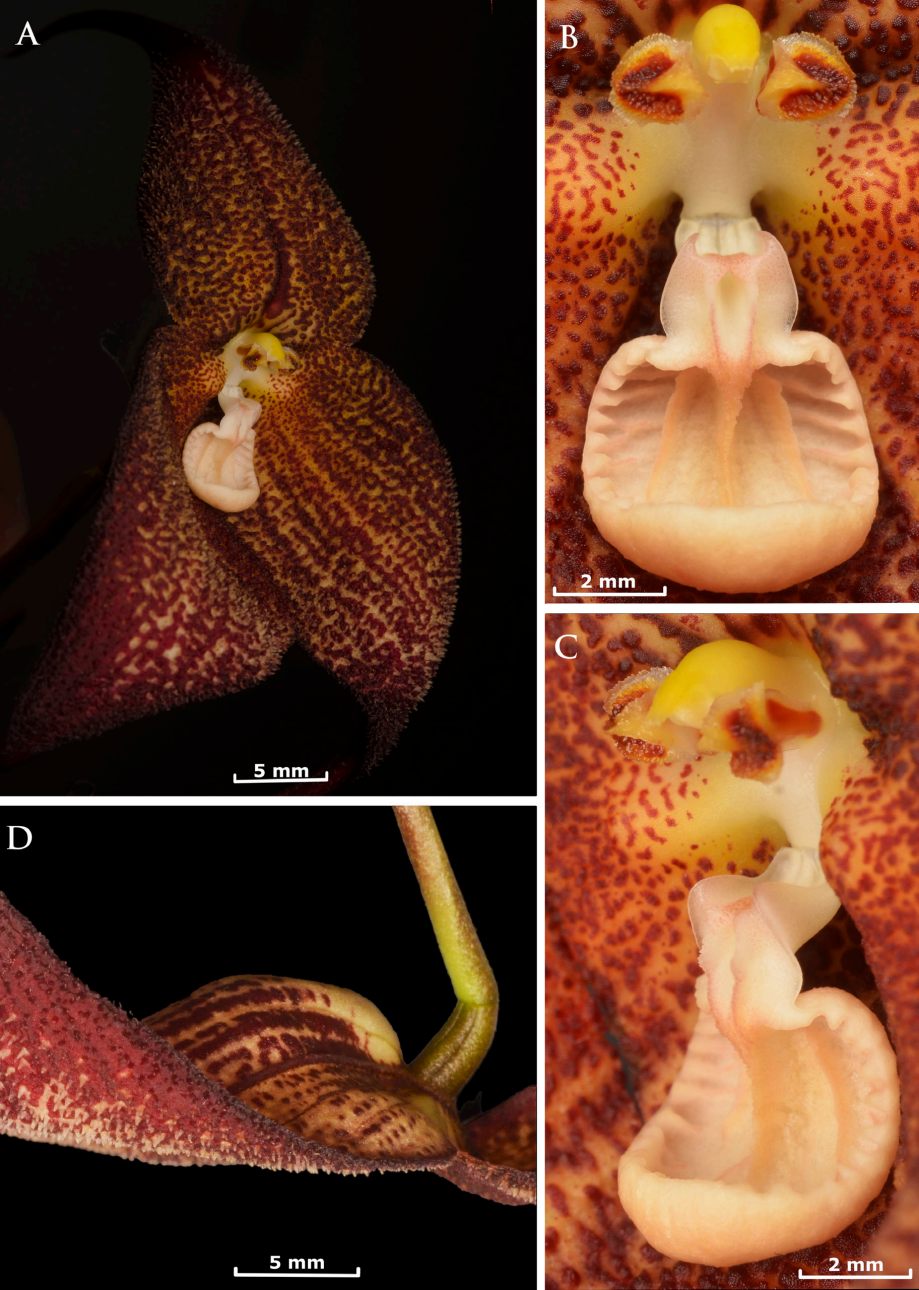
*Dracula aguilarii* Baquero, M.M. Jiménez & Iturralde in vivo. **A**. Flower in ¾ view, showing the deep center, the convex sepals toward the apex, the short pubescence, and the proportionally small lip in relation to the sepals; **B**. Close-up of the petals and lip, frontal view; **C**. Close-up of the column, petals, and lip, lateral view; **D**. Side view of the flower, the deep and large mentum is visible as well as the convex towards the apex and pubescent sepals. Photographs by Luis E. Baquero (*LB 3142*, holotype, QCNE).

**Figure 3.**
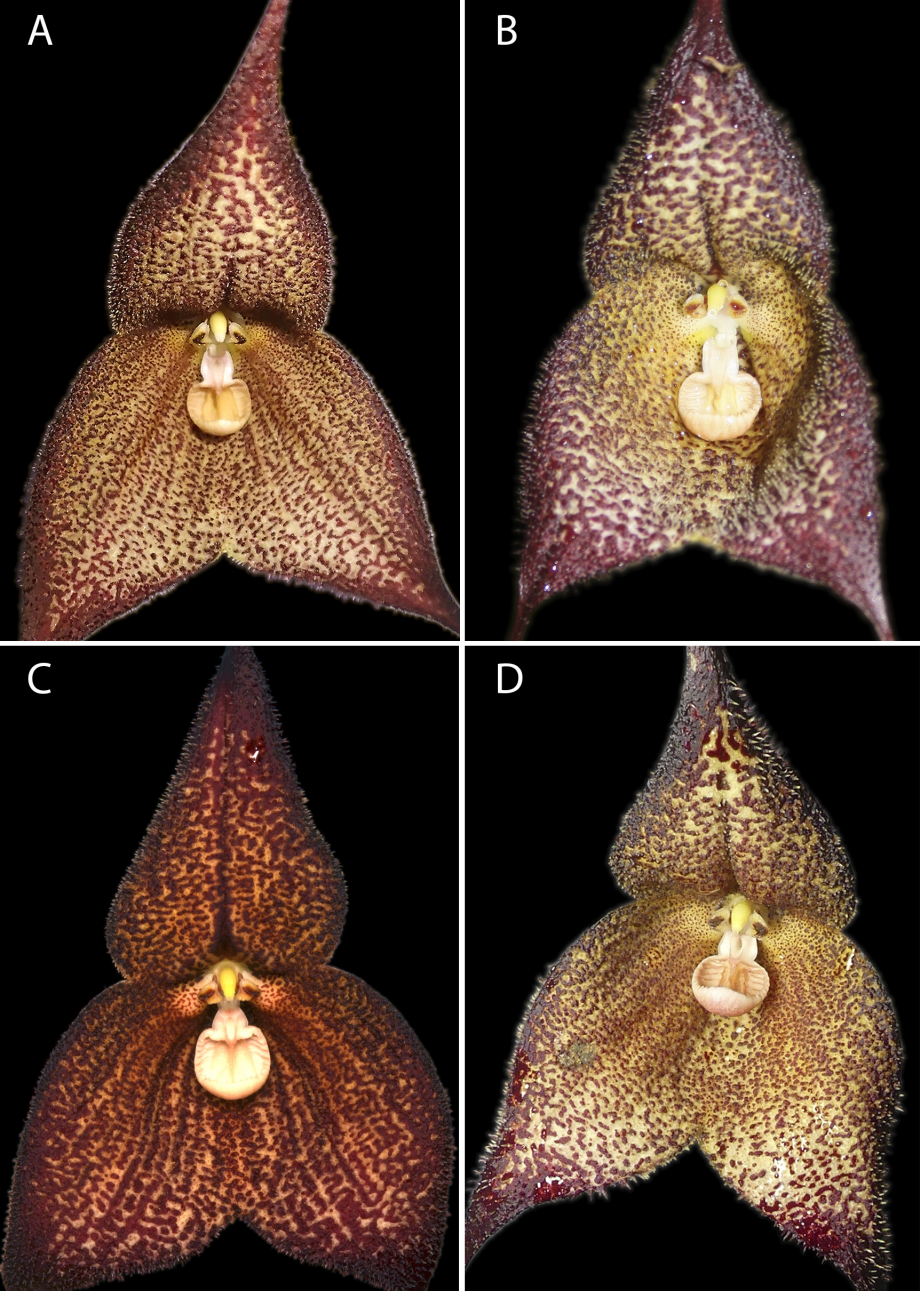
Colour variation in *Dracula aguilarii* Baquero, M.M. Jiménez & Iturralde. **A**. Photo by Mario Aguilar from near Piñas; **B**. Photo by Marco M. Jiménez from a plant in situ at the southernmost known population near Balsas; **C**. Photo by Luis E Baquero from Holotype, *LB 3142*, QCNE (near Balsas); **D**. Photo by Marco M. Jiménez from a cultivated plant collected near Balsas.

*Dracula aguilarii* is endemic to El Oro Province, southwestern Ecuador, where it occurs at 900–1200 m elevation. In contrast, *D. hirtzii* is distributed in Ecuador and Colombia at 1500–2100 m, whereas *D. ligiae* (c. 2050 m) and *D. roezlii* (1800–2300 m) are restricted to Colombia. The new species occurs at substantially lower elevations and is geographically separated by approximately 350 km from the southernmost known population of *D. hirtzii*. Among all species of *Dracula*, *D. aguilarii* is immediately recognizable by its large flowers with a deep, broad, rounded sepaline cup. In other large-flowered species of the genus, the sepaline cup is typically formed by a narrow, longitudinal mentum beneath the lip. Although several species combine the mentum with sepal concavities (i.e., *D. pholeodytes* Luer & R. Escobar, *D. benedictii* (Reichenbach) Luer, *D. smaug* Baquero & Meyer, G., *D. vampira* (Luer) Luer, *D. roezlii*, *D. ligiae*), the rounded, deeply concave synsepal of *D. aguilarii* contrasts conspicuously with the convex shape of the sepals toward the apex (Figs 1, 2, 3). Furthermore, the lip of *D. aguilarii* is proportionately much smaller relative to sepal length and width, creating the impression of a disproportionately reduced lip compared to the synsepal and to other large-flowered species (Luer 1993, 1994, 2002, 2005, 2009; Luer and Thoerle 2010, 2012) (Figs 2, 3).

**Figure 4.**
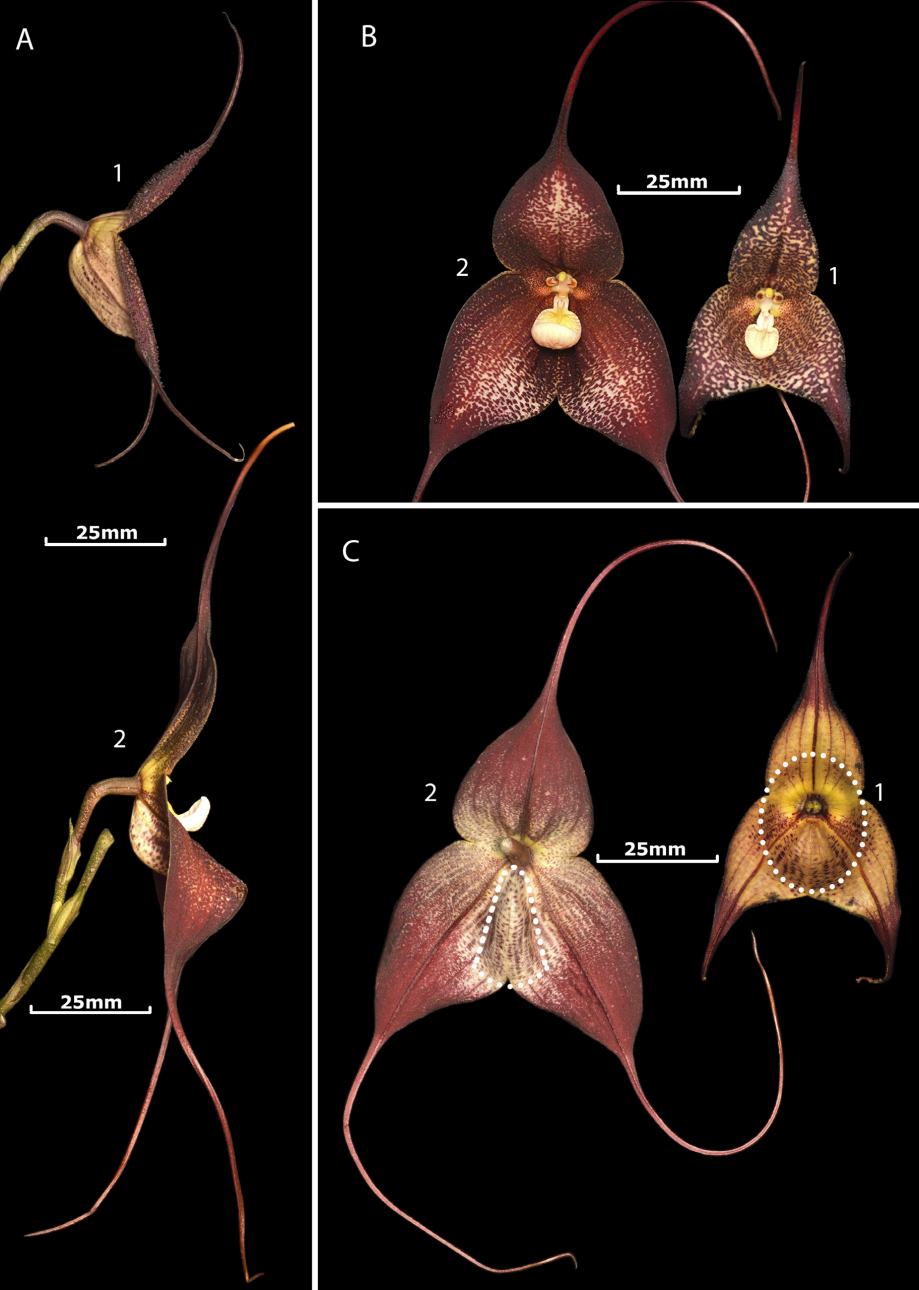
Comparison of *Dracula aguilarii* Baquero, M.M. Jiménez & Iturralde vs. *D. hirtzii* Luer. **A**. Side view of the flowers; **A1**. *Dracula aguilarii*; **A2**. *Dracula hirtzii*; **B**. Adaxial (frontal) view of the flowers; **B1**. *Dracula aguilarii*; **B2**. *Dracula hirtzii*; **C**. Abaxial (back) view of the flowers; **C1**. *Dracula aguilarii*, white dotted lines highlight the broad, rounded, concave sepaline cup; **C2**. *Dracula hirtzii*, the white dotted lines highlight the narrow, longitudinal mentum. Photographs by Luis E. Baquero from *Dracula aguilarii*, *LB 3143*, (QCNE), and *Dracula hirtzii*, *LB 3144* (QCNE).

**Figure 5.**
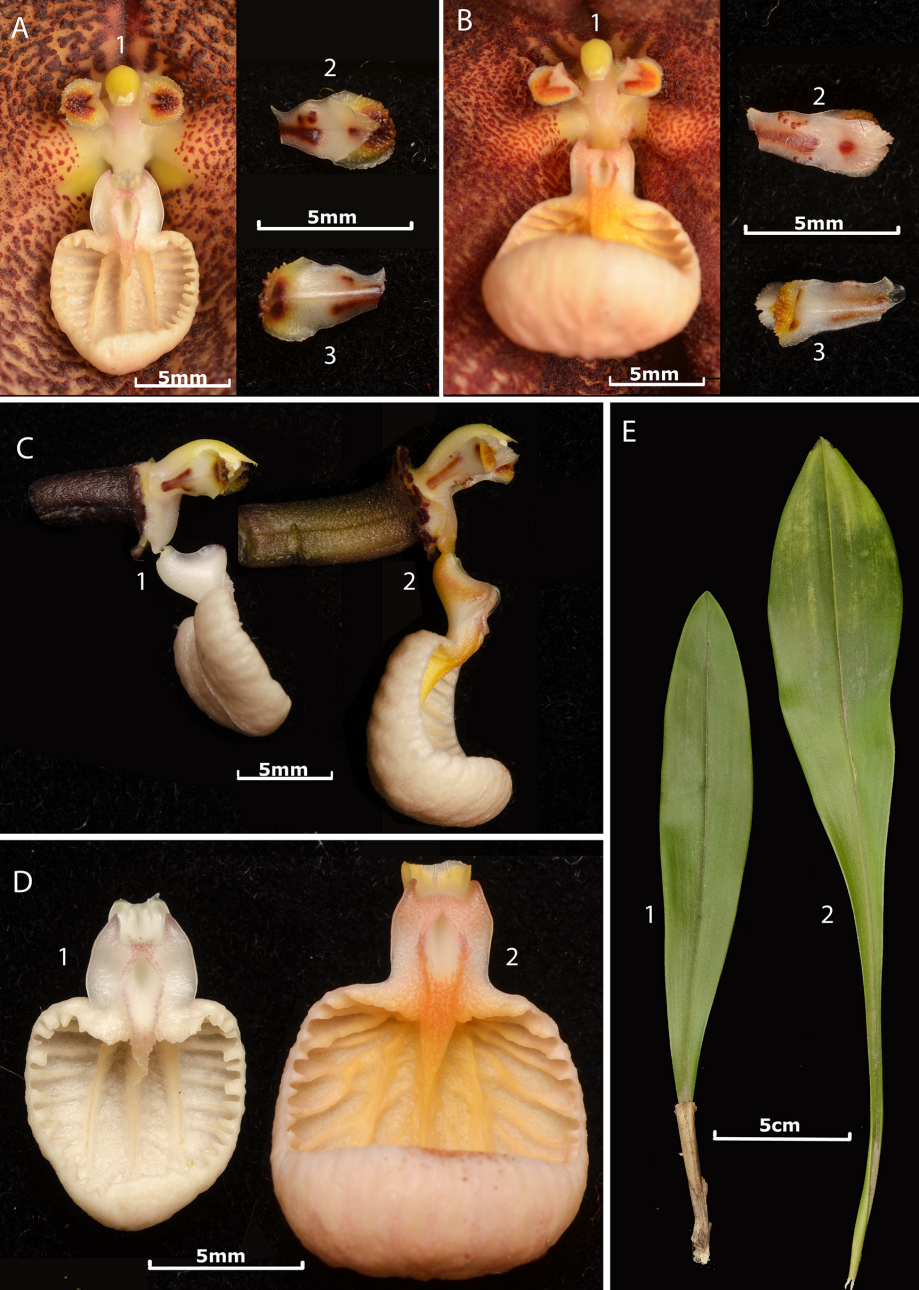
Comparison of *Dracula aguilarii* Baquero, M.M. Jimenez & Iturralde vs. *D. hirtzii* Luer. **A**. *Dracula aguilarii*; **A1**. Frontal view and close-up of the column, petals, and lip; **A2**. Adaxial view of the petal; **A3**. Abaxial view of the petal; **B**. *Dracula hirtzii*; **B1**. Frontal view and close-up of the column, petals and lip; **B2**. Adaxial view of the petal; **B3**. Abaxial view of the petal; **C**. Lateral view of the column, petals and lip; **C1**. *Dracula aguilarii*; **C2**. *Dracula hirtzii*; **D**. Adaxial view of the lip; **D1**. *Dracula aguilarii*; **D2**. *Dracula hirtzii*; **E**. Adaxial view of the leaves; **E1**. *Dracula aguilarii*; **E2**. *Dracula hirtzii*. Photographs by Luis E. Baquero.

**Figure 6.**
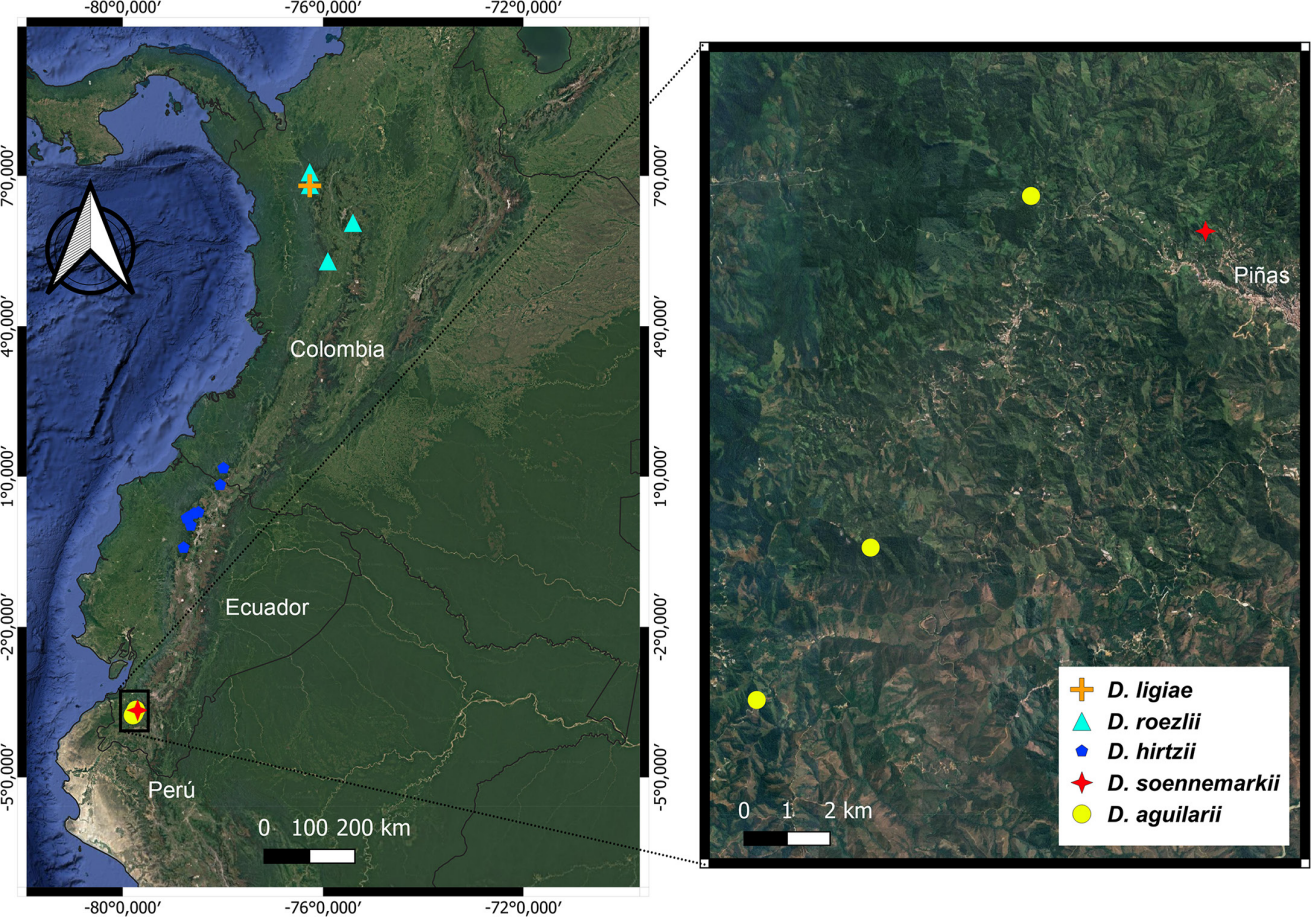
Distribution of the known populations of *Dracula aguilarii* Baquero, M.M. Jiménez & Iturralde and morphologically similar species, and confirmed locality of *D. soennemarkii* Luer & Dalström, in the northern Andes. Base map layer was obtained from Google Satellite imagery (Google LLC; https://www.google.com/earth/).

**Figure 7.**
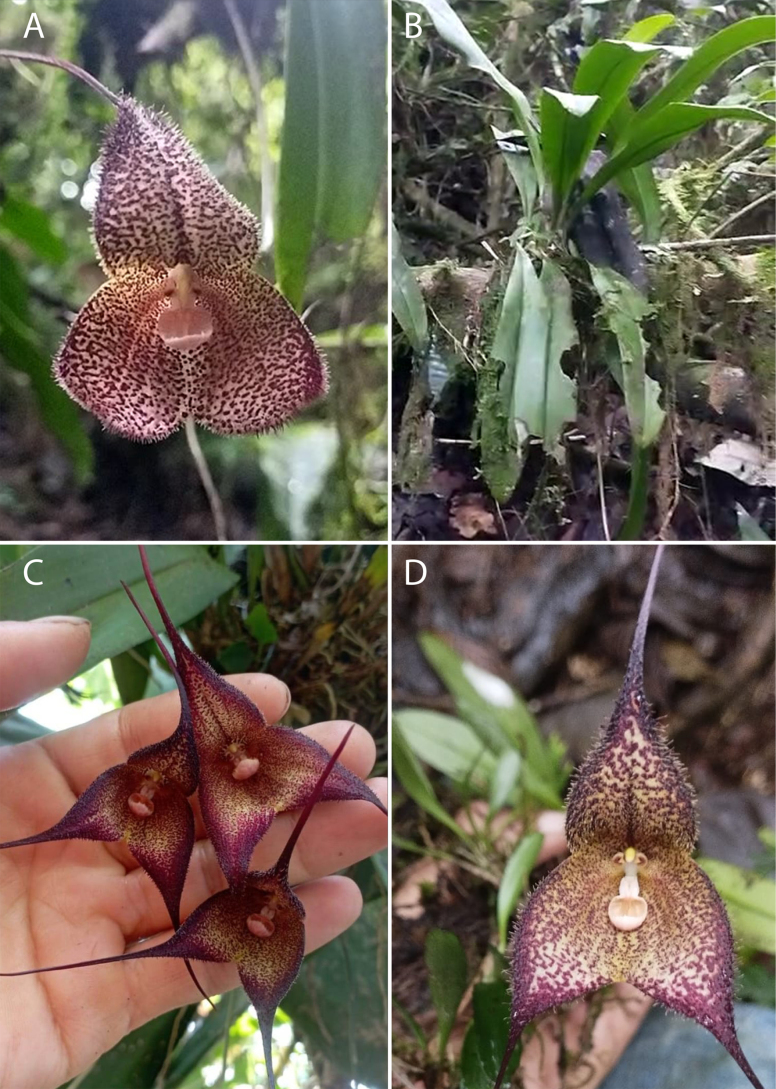
*Dracula aguilarii* Baquero, M.M. Jimenez & Iturralde. **A, B**. Flower and plant in situ near El Caucho; **C, D**. Photographs of other plants in cultivation. Photographs by José Bustamante.

Six flowering individuals were examined, four of which were photographed. Minor variation was observed in sepal pubescence length and spot coloration, ranging from nearly blood-red to dark reddish-brown. In contrast, the shape and size of the sepaline cup and lip were consistent across all examined individuals (Fig. 3).

**Figure 8.**
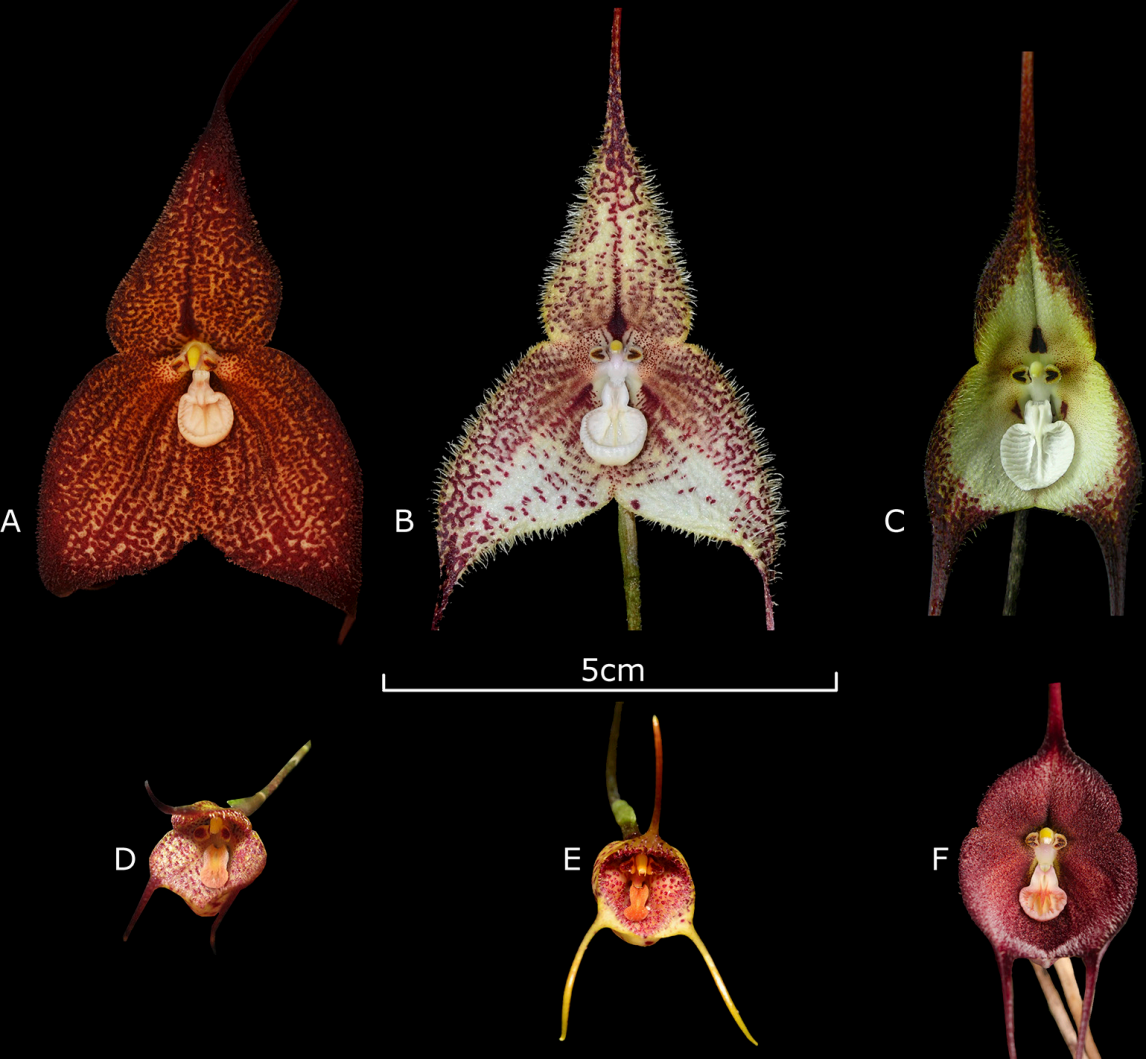
Known species and natural hybrids of *Dracula* from El Oro province, southwestern Ecuador. **A**. *D. aguilarii* Baquero, M.M. Jiménez & Iturralde; **B**. *D. woolwardiae* (F. Lehmann ex Kraenzlin) Luer; **C**. *D. cordobae* Luer; **D**. *D. mopsus* (F. Lehmann & Kraenzlin) Luer; **E**. *D. × pinasensis* Zambrano & Solano; **F**. *D. soennemarkii* Luer & Dalström. **A**. From holotype, *LB 3142*QCNE, and **B–F**. From near Piñas. Photographs by Luis E. Baquero (**A–E**), and Mario Aguilar (**F**).

*Dracula aguilarii* occurs in sympatry with *D. cordobae* and *D. woolwardiae*, *D. mopsus* and *D. × pinasensis*. *Dracula soennemarkii* has also been recorded in nearby localities (Fig. 8). The discovery of *D. aguilarii* in the cloud forests of El Oro province raises to six the number of *Dracula* species recorded in southwestern Ecuador. The confirmed locality of *D. soennemarkii* represents a significant taxonomic and biogeographic update. Because its original description lacked georeferenced data, its rediscovery in El Oro provides essential information regarding its habitat preferences and elevational range.

From a conservation perspective, both *D. aguilarii* and *D. soennemarkii* face a high risk of extinction. Their extremely restricted distributions, small known population sizes, and rapid habitat degradation in El Oro qualify them as Endangered and Critically Endangered, respectively, under IUCN (2024) criteria. Ongoing expansion of cattle ranching, selective logging, and fragmentation of forest remnants have resulted in the near disappearance of suitable habitat. These pressures are exacerbated by the illegal collection of ornamental orchids, including species of *Dracula*, for international trade.

## Supplementary Material

XML Treatment for Dracula aguilarii

XML Treatment for Dracula soennemarkii

## Additional information

### Use of AI

AI was used for English language editing. All text was carefully reviewed and approved by the authors.

### Author ORCIDs

Luis E. Baquero R. https://orcid.org/0000-0002-1444-5727

Marco M. Jiménez https://orcid.org/0000-0002-9502-5651

Gabriel A. Iturralde https://orcid.org/0000-0003-2456-0929

### Data availability

All of the data that support the findings of this study are available in the main text.

## References

[B1] Bachman S, Moat J, Hill A, de la Torre J, Scott B (2011) Supporting Red List threat assessments with GeoCAT: geospatial conservation assessment tool. ZooKeys 150: 117–126. 10.3897/zookeys.150.2109 PMC3234434 22207809

[B2] Baquero L (2013) *Dracula marinii* Baquero, recently discovered Ecuadorian species of orchid in the Pleurothallidinae (Orchidaceae). Orquideología 30(2): 1–67.

[B3] Baquero L, Meyer G (2014) *Dracula smaug*, Baquero & Gary Mey., una especie de orquídea ecuatoriana recientemente descubierta en la subtribu Pleurothallidinae. Orquideología 31(2): 84–93.

[B4] Baquero L, Domínguez E, Mesa S, Parra-Sánchez E (2025) *Dracula colombiana* (Pleurothallidinae: Orchidaceae), a new orchid species with a history of misidentification in trade and collections. Phytotaxa 706(1): 081–090. 10.11646/phytotaxa.706.1.6

[B5] Beentje H (2016) The Kew Plant Glossary: An Illustrated Dictionary of Plant Terms (2^nd^ Edn.). Kew Publishing, Royal Botanical Gardens, Richmond, 184 pp. 10.2307/4110976

[B6] Cavestro W (2013) Une nouvelle espèce de *Dracula* d’Équateur: *Dracula fernandezii* Cavestro. Rhone-Alpes-Orchidée 51: 10–14. https://www.orchid-nord.com/~~~PROTECTED_TN_158~~~/~~~PROTECTED_TN_159~~~%20fernandezii/dracula_cavestro_n51.pdf

[B7] Cavestro W, Fernández J (2016) *Dracula marieae*: une nouvelle espèce du nord de l’Équateur. L’Orchidophile 208: 57–64.

[B8] Doucette A (2011) *Dracula immunda* (Orchidaceae: Pleurothallidinae), a new species from Panama. Phytotaxa 16(1): 37–44. 10.11646/phytotaxa.16.1.2

[B9] Doucette A (2012) *Dracula agnosia* (Orchidaceae: Pleurothallidinae), a long confused undescribed species. Phytotaxa 56(1): 23–27. 10.11646/phytotaxa.56.1.6

[B10] Endara L, Grimaldi DA, Roy BA (2010) Lord of the flies: pollination of *Dracula* orchids. Lankesteriana 10(1): 1–11. 10.15517/lank.v10i1.18318

[B11] Faria HM, Karremans AP, Baquero L, Gil-Amaya K, Cerna M, Pessoa EM (2025) Unidirectional west-to-east trans-Andean dispersals characterize the biogeography of *Dracula* Luer (Pleurothallidinae-Orchidaceae). Botanical Journal of the Linnean Society 212(4): 1–10. 10.1093/botlinnean/boaf113

[B12] GAD El ORO (2019) Actualización integral del plan de desarrollo y ordenamiento territorial de la provincia de El Oro. Gobierno Autónomo Descentralizado Provincia de El Oro. https://multimedia.planificacion.gob.ec/PDOT/descargas.html

[B13] Gil-Amaya K, Fernández M, Oses L, Benavides-Acevedo M, Grimaldi D, Blanco MA, Karremans AP (2025) Pollination ecology of *Dracula erythrochaete* (Orchidaceae): brood-site imitation or food deception?. Botanical Journal of the Linnean Society 207: 279–297. 10.1093/botlinnean/boae054

[B14] Hermes C, Jansen J, Schaefer H (2017) Habitat requirements and population estimate of the endangered Ecuadorian Tapaculo *Scytalopus robbinsi*. Bird Conservation International 28(2): 302–318. 10.1017/s095927091600054x

[B15] IUCN (2024) Guidelines for Using the IUCN Red List Categories and Criteria. Version 16. Prepared by the Standards and Petitions Committee. https://www.iucnredlist.org/documents/RedListGuidelines.pdf

[B16] Jørgensen PM, León-Yánez S [Eds] (1999) Catalogue of the Vascular Plants of Ecuador. Missouri Botanical Garden Press, Saint Louis, 1181 pp.

[B17] Luer C (1978) *Dracula*, a new genus in the Pleurothallidinae. Selbyana 2: 190–198.

[B18] Luer C (1986) Icones Pleurothallidinarum I: Systematics of the Pleurothallidinae. Monographs in systematic botany from the Missouri Botanical Garden 15: 1–78. 10.5962/bhl.title.149317

[B19] Luer C (1993) Icones Pleurothallidinarum X: Systematics of *Dracula*. Monographs in systematic botany from the Missouri Botanical Garden 46: 1–244. 10.5962/bhl.title.149317

[B20] Luer C (1994) Icones Pleurothallidinarum XI: Systematics of *Lepanthes* Subgenus *Brachycladium* and Pleurothallis subgenus *Aenigma*, subgenus *Elongatia*, subgenus *Kraenzlinella*. Addenda to *Dracula*, *Lepanthopsis*, *Myoxanthus*, *Platystele*, *Porroglossum* and *Trisetella*. Monographs in systematic botany from the Missouri Botanical Garden 52: 1–137.

[B21] Luer C (2002) Icones Pleurothallidinarum XXIV: A first century of new species of *Stelis* of Ecuador, part one. Addenda to *Barbosella*, *Dracula*, *Dresslerella*, *Lepanthopsis*, *Platystele*, *Pleurothallis*, *Restrepia*, *Scaphosepalum*, *Teagueia* and *Trichosalpinx*. Monographs in systematics botany from the Missouri Botanical Garden 88: 1–122.

[B22] Luer C (2005) Icones Pleurothallidinarum XXVII: *Dryadella* and *Acronia* section Macrophyllae-Fasciculatae. Addenda to *Acianthera*, *Andinia*, *Dracula*, *Dresslerella*, *Lepanthes* and *Restrepia*. Monographs in systematic botany from the Missouri Botanical Garden 103: 1–311. 10.5962/bhl.title.149317

[B23] Luer C (2009) Miscellaneous new species in the Pleurothallidinae (Orchidaceae). Selbyana 30: 19–61. 10.5962/bhl.part.14732

[B24] Luer C, Thoerle L (2010) *Dracula*. In: Dodson C, Luer C (Eds) Flora of Ecuador. Orchidaceae: Genera *Cyrtochiloides*-*Epibator*. Department of Plant and Environmental Sciences, University of Gothenburg, Göteborg.

[B25] Luer C, Thoerle L (2012) Miscellaneous new species in the Pleurothallidinae (Orchidaceae). Harvard Papers in Botany 17(2): 333–368. 10.3100/025.017.0214

[B26] Meyer G, Cameron K (2009) A preliminary phylogenetic study of *Dracula* (Pleurothallidinae, Epidendroideae, Orchidaceae) based on plastid *matk* sequence data. In: Pridgeon AM, Suarez JP (Eds) Proceedings of the Second Scientific Conference on Andean Orchids, Loja, Ecuador, 100–114.

[B27] Meyer G, Baquero L, Cameron K (2012) A new Ecuadorian species of *Dracula*: Pleurothallidinae (Orchidaceae). Orchideenjournal 19: e107.

[B28] Parra-Sanchez E, Baquero L (2023) Circumscription, first confirmed locality, and conservation status of *Dracula anthracina* (Orchidaceae). Systematic Botany 48(2): 200–207. 10.1600/036364423X16847773873080

[B29] Peláez N, Meyer G, Rendon-Jaramillo U, Fernández JD, López-Álvarez N, Mazariegos HL (2020) *Dracula irmelinae*, a new species in the subtribe Pleurothallidinae (Orchidaceae) from the Western Andes of Colombia. Lankesteriana 20: 137–149. 10.15517/lank.v20i2.41823

[B30] POWO (2026) Plants of the World Online. Facilitated by the Royal Botanic Gardens, Kew. https://powo.science.kew.org/ [accessed 24.02.2026]

[B31] Pridgeon AM, Solano R, Chase MW (2001) Phylogenetic relationships in Pleurothallidinae (Orchidaceae): combined evidence from nuclear and plastid DNA sequences. American Journal of Botany 88(12): 2286–2308. 10.2307/3558390 21669661

[B32] Yánez-Muñoz M, Medina J, Garzón-Santomaro C (2019) Capítulo I: Aspectos generales para el estudio de anfibios, reptiles y aves de la provincia de El Oro. In: Garzón-Santomaro C, Sánchez-Nivicela JC, Mena-Valenzuela P, González-Romero D, Mena-Jaén JL (Eds) Anfibios, Reptiles y Aves de la Provincia de El Oro. Una Guía Para la Identificación de Especies del Páramo al Manglar (2^da^ Edn.). Gadpeoinabio, Quito.

[B33] Yeager J, Baquero LE, Zarling A (2020) Mediating ethical considerations in the conservation and sustainable biocommerce of the jewels of the rainforest. Journal for Nature Conservation 54: e125803. 10.1016/j.jnc.2020.125803

